# Comparison of acid‐lowering drugs for endoscopy negative reflux disease: Systematic review and network Meta‐Analysis

**DOI:** 10.1111/nmo.14469

**Published:** 2022-09-25

**Authors:** Brigida Barberio, Pierfrancesco Visaggi, Edoardo Savarino, Nicola de Bortoli, Christopher J. Black, Alexander C. Ford

**Affiliations:** ^1^ Division of Gastroenterology Department of Surgery, Oncology and Gastroenterology University of Padua Padua Italy; ^2^ Gastroenterology Unit Department of Translational Research and New Technologies in Medicine and Surgery University of Pisa Pisa Italy; ^3^ Leeds Gastroenterology Institute St. James's University Hospital Leeds UK; ^4^ Leeds Institute of Medical Research at St. James's University of Leeds Leeds UK

**Keywords:** alginate, endoscopy‐negative reflux disease, histamine‐2‐receptor antagonist, potassium‐competitive acid blocker, proton pump inhibitor

## Abstract

**Background:**

The comparative efficacy and safety of medical therapies for gastro‐esophageal reflux symptoms in endoscopy‐negative reflux disease is unclear. We conducted a network meta‐analysis to evaluate efficacy and safety of proton pump inhibitors (PPIs), histamine‐2‐receptor antagonists, potassium‐competitive acid blockers (PCABs), and alginates in patients with endoscopy‐negative reflux disease.

**Methods:**

We searched MEDLINE, EMBASE, EMBASE Classic, and the Cochrane central register of controlled trials from inception to February 1, 2022. We included randomized controlled trials (RCTs) comparing efficacy of all drugs versus each other, or versus a placebo, in adults with endoscopy‐negative reflux disease. Results were reported as pooled relative risks with 95% confidence intervals to summarize effect of each comparison tested, with treatments ranked according to *P*‐score.

**Key Results:**

We identified 23 RCTs containing 10,735 subjects with endoscopy‐negative reflux disease. Based on failure to achieve complete relief of symptoms between ≥2 and <4 weeks, omeprazole 20 mg o.d. (*P*‐score 0.94) ranked first, with esomeprazole 20 mg o.d. or 40 mg o.d. ranked second and third. In achieving adequate relief, only rabeprazole 10 mg o.d. was significantly more efficacious than placebo. For failure to achieve complete relief at ≥4 weeks, dexlansoprazole 30 mg o.d. (*P*‐score 0.95) ranked first, with 30 ml alginate q.i.d. combined with omeprazole 20 mg o.d., and 30 ml alginate t.i.d. second and third. In terms of failure to achieve adequate relief at ≥4 weeks, dexlansoprazole 60 mg o.d. ranked first (*P*‐score 0.90), with dexlansoprazole 30 mg o.d. and rabeprazole 20 mg o.d. second and third. All drugs were safe and well‐tolerated.

**Conclusions & Inferences:**

Our results confirm superiority of PPIs compared with most other drugs in treating endoscopy‐negative reflux disease. Future RCTs should aim to better classify patients with endoscopy‐negative reflux disease, and to establish the role of alginates and PCABs in achieving symptom relief in both the short‐ and long‐term.

## Graphical abstract

Our network demonstrated that, based on failure to achieve complete relief of symptoms between ≥2 and <4 weeks, omeprazole 20 mg o.d. ranked first, with esomeprazole 20 mg o.d. or 40 mg o.d. ranked second and third. For failure to achieve complete relief at ≥4 weeks, dexlansoprazole 30 mg o.d. ranked first, with 30 ml alginate q.i.d. combined with omeprazole 20 mg o.d., and 30 ml alginate t.i.d. second and third.

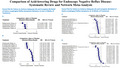

**Abbreviations**
CI: confidence interval
EE: erosive esophagitis
GERD: gastro‐esophageal reflux disease
H_2_RA: histamine‐2‐receptor antagonist
PCAB: potassium‐competitive acid blocker
PPI: proton pump inhibitor
RCT: randomized controlled trial
RR: relative risk

1

**Key points**

1. Patients with erosive esophagitis (EE) represent only a minority of those with gastro‐esophageal reflux disease (GERD), with many having endoscopy negative reflux disease.
2. Proton pump inhibitors (PPIs), histamine‐2‐receptor antagonists (H2RAs), potassium‐competitive acid blockers (PCABs), and alginates are effective for GERD. However, there are uncertainties in terms of best choice of drug to treat patients with endoscopy negative reflux disease, as most studies have focused on patients EE.
3. Our network meta‐analysis is, to our knowledge, the first to report efficacy and safety of PPIs, H2RAs, PCABs, and alginates compared with each other or with placebo in patients with endoscopy negative reflux disease.
4. In the short‐term, the PPIs omeprazole and esomeprazole were the best treatments in terms of achieving complete relief of symptoms, and rabeprazole was best for adequate relief.
5. In the longer term, dexlansoprazole, alginate combined with omeprazole, and alginate alone ranked first, second, and third for complete relief of symptoms, with dexlansoprazole and rabeprazole most efficacious for adequate relief.

## 2 INTRODUCTION

Gastro‐esophageal reflux symptoms are common and usually chronic. Although some reflux of stomach contents into the esophagus, with or without symptoms, is physiological, gastro‐esophageal reflux disease (GERD) is a condition that develops when the reflux of stomach contents causes troublesome symptoms and/or complications.^1^ These symptoms include typical ones, such as heartburn, regurgitation, or both, angina‐mimicking non‐cardiac chest pain, and extra‐esophageal symptoms, including chronic cough or laryngitis.^2^, ^3^ Esophageal stricture, Barrett's esophagus, and esophageal adenocarcinoma are some of the potential complications of GERD.^4^, ^5^, ^6^, ^7^ The prevalence of GERD based on symptom reporting in individual cross‐sectional surveys varies strikingly from 2.5% to over 52%, according to geographical location,^8^ but is much lower in Asia than in Western countries.^8^, ^9^, ^10^

Based on the presence or absence of mucosal damage, GERD patients can be subclassified as to whether there is evidence of erosive esophagitis (EE) or not.^11^, ^12^ However, patients with EE represent only a minority of those with GERD, estimated at 30%, with the remaining 70% having endoscopy‐negative reflux disease with increased esophageal acid exposure, reflux hypersensitivity, or functional heartburn.^1^, ^12^, ^13^ pH monitoring and impedance testing are needed to distinguish between the latter three conditions.^1^, ^12^ In fact, a diagnosis of endoscopy‐negative reflux disease is confirmed by objective evidence that symptoms are related to reflux, on the basis of abnormal esophageal acid exposure, while reflux hypersensitivity is defined by a positive correlation between occurrence of symptoms and episodes of reflux during pH monitoring.^12^, ^14^ The impact of GERD on quality of life and social functioning is substantial, irrespective of whether the patient has EE or endoscopy‐negative reflux disease.^15^ In fact, disease‐specific symptom scores and generic quality of life scores in patients with EE and endoscopy‐negative reflux disease are similar, and lower than in healthy controls.^16^ Treatment with efficacious drugs that provide rapid relief of symptoms and a reduction in the number and severity of reflux episodes, as well as improving quality of life is, therefore, important.^17^

Proton pump inhibitors (PPIs) have been used widely to manage GERD, due to their powerful inhibition of gastric acid secretion.^17^ However, there are controversies regarding their efficacy in patients with endoscopy‐negative reflux disease. A previous systematic review demonstrated that the rate of therapeutic response to PPIs after 4 weeks of treatment was lower in patients with endoscopy‐negative reflux disease, compared with those with EE, by a factor of about 20%.^18^ Nevertheless, a Cochrane review demonstrated superiority for PPI therapy, in terms of relief of heartburn in endoscopy‐negative reflux disease, compared with histamine‐2‐receptor antagonists (H_2_RAs) or prokinetics.^19^ More recently, the novel drug class of potassium‐competitive acid blockers (PCABs) have been compared with other acid‐suppressive treatments in endoscopy‐negative reflux disease, although with conflicting results.^20^, ^21^ Finally, alginate‐based therapy, with its raft‐forming properties, may also be beneficial in GERD, as demonstrated by various studies.^22^, ^23^, ^24^ In patients with endoscopy‐negative reflux disease, evidence suggests that alginates improve symptoms either as a monotherapy or when combined with PPIs as add‐on therapy.^22^, ^25^

There are, however, considerable uncertainties in terms of choice of drug to treat patients with endoscopy‐negative reflux disease, compounded by the fact that most studies of drug therapy for GERD have focused their attention on patients with EE.^17^ Further complicating the situation is the fact that there are few head‐to‐head trials comparing different acid‐suppressive drugs or alginate‐based compounds in patients with endoscopy‐negative reflux disease. In this scenario, network meta‐analysis may be useful, because the methodology employed allows indirect, as well as direct, comparisons to be made across different randomized controlled trials (RCTs), increasing the number of participants' data available for analysis. In addition, this technique allows the development of a credible ranking system for the likely efficacy of different drugs, which can aid clinical decision‐making. We, therefore, performed a network meta‐analysis to evaluate PPIs, H_2_RAs, PCABs, and alginates compared with each other or with placebo in terms of their efficacy and safety in patients with endoscopy‐negative reflux disease.

## 3 METHODS

### 3.1 Search strategy and study selection

For this systematic review and network meta‐analysis, we searched MEDLINE (1946 to February 1, 2022), EMBASE and EMBASE classic (1947 to February 1, 2022), and the Cochrane central register of controlled trials (from 2005 to February 1, 2022). We also hand‐searched conference proceedings (Digestive Diseases Week, American College of Gastroenterology, United European Gastroenterology Week, and the Asian Pacific Digestive Week) between 2006 and February 2022 to identify studies published only in abstract form.

RCTs examining the efficacy of PPIs, H_2_RAs, PCABs, or alginates versus each other, or versus a placebo, in adult patients (>90% of participants over the age of 16 years) with endoscopy‐negative reflux disease were eligible for inclusion (Table 1). Endoscopy‐negative reflux disease was defined as the presence of heartburn and/or regurgitation and a normal upper endoscopy, with or without positive pH monitoring and impedance testing of ≥24 hours duration, performed off anti‐reflux medications. The first period of crossover RCTs were also eligible for inclusion. Duration of therapy had to be at least 2 weeks. Trials using any dose or combination of PPIs, H_2_RAs, PCABs, or alginates were eligible. Studies had to report either an assessment of failure to achieve complete relief of gastro‐esophageal reflux symptoms (heartburn and/or regurgitation), or failure to achieve adequate relief of gastro‐esophageal reflux symptoms, in patients with endoscopy‐negative reflux disease. Complete relief in individual RCTs was defined as the absence of heartburn and/or regurgitation, where reported, and adequate relief of heartburn and/or regurgitation was defined according to various criteria (Table S1). Ethical approval for this evidence synthesis was not required.

**TABLE 1:** Eligibility criteria

Randomized controlled trials
Patients ≥16 years diagnosed with non‐erosive reflux disease^a^
Compared proton pump inhibitors, histamine‐2‐receptor antagonist, potassium‐competitive acid blockers, or alginates with each other, or with placebo
Minimum duration of therapy of 2 weeks
Assessment of failure of complete relief of gastro‐esophageal reflux symptoms (heartburn and/or regurgitation) or adequate relief of gastro‐esophageal reflux symptoms at last time point of assessment in the trial

^a^ Defined as the presence of heartburn and/or regurgitation and normal upper endoscopy, with or without positive pH monitoring and impedance testing of ≥24 hours of duration, performed off anti‐reflux medications.

Studies were identified with the terms non‐erosive reflux disease or NERD or endoscopy‐negative reflux disease or symptomatic reflux disease (all as medical subject headings and as free text terms). These were combined using the set operator AND with studies identified with the terms: proton‐pump inhibitor, PPI, pantoprazole, omeprazole, esomeprazole, lansoprazole, dexlansoprazole, rabeprazole, potassium‐competitive acid blocker, PCAB, K‐CAB, vonoprazan, tegoprazan, fexuprazan, revaprazan, histamine‐receptor antagonist, H2‐RA, H2RA, ranitidine, cimetidine, roxatidine, famotidine, nizatidine, alginate, or sodium alginate. There were no language restrictions. We screened the titles and abstracts of all citations identified by our search for potential suitability and retrieved those that appeared relevant to examine them in more detail. We performed a recursive search, using the bibliographies of all eligible articles. We translated foreign language articles, where required. If a study appeared potentially eligible, but did not report the data required, we planned to contact authors to obtain Data S1. We performed eligibility assessment independently. This was done by two investigators (BB and PV), using pre‐designed eligibility forms. We resolved any disagreements by consensus and measured the degree of agreement with a kappa statistic.

### 3.2 Outcome assessment

The primary outcome assessed was the efficacy of PPIs, H_2_RAs, PCABs, and alginates versus each other, or placebo, in terms of failure to achieve complete relief of gastro‐esophageal reflux symptoms in patients with endoscopy‐negative reflux disease. Secondary outcomes included failure to achieve adequate relief of gastro‐esophageal reflux symptoms and treatment‐related adverse events.

### 3.3 Data extraction

Data were extracted independently by two investigators (BB, PV) on to a Microsoft Excel spreadsheet (XP professional edition; Microsoft, Redmond, WA) as dichotomous outcomes (complete relief of symptoms or no complete relief of symptoms, and adequate relief or no adequate relief). Two investigators (BB, PV) extracted all trial data independently, with results of data extraction compared and any disagreements resolved by consensus. We extracted the following clinical data for each trial, where available: year of publication, country of origin, number of centers, sample size, endpoint(s) of the study, type and dosage of treatments, duration of treatments, and number of individuals incurring each (or any) of the adverse events. Wherever trial reporting allowed, we extracted data as intention‐to‐treat analyses, with all dropouts assumed to be treatment failures (i.e., failed to achieve complete relief of symptoms or failed to achieve adequate relief of symptoms). If the number of dropouts was not clear from the original article, we extracted data only for patients with reported evaluable data. Individual trials reported these data at different timepoints, but we standardized this by extracting data either between ≥2 and <4 weeks of treatment or ≥4 weeks of treatment.

### 3.4 Quality assessment and risk of bias

We used the Cochrane Risk of Bias tool to assess the quality of studies.^26^ Two investigators (BB and PV) assessed study quality independently, with disagreements resolved by discussion. For all RCTs, we recorded the method used to generate the randomization schedule and conceal treatment allocation, whether participants, personnel, and outcome assessors were blinded, whether there was evidence of incomplete patient outcome data, and whether there was evidence of selective reporting of patient outcomes.

### 3.5 Data synthesis and statistical analysis

We performed a network meta‐analysis using the frequentist model with the statistical package netmeta (version 0.9–0), in R (version 3.4.6) to compare (directly and indirectly) the efficacy and safety of each of the treatments of interest across studies. We reported this according to the Preferred reporting Items for Systematic Reviews and Meta‐Analyses (PRISMA) extension statement for network meta‐analyses,^27^ to explore direct and indirect treatment comparisons on the efficacy and safety of each intervention. Network meta‐analysis usually give a more precise estimate of the relative efficacy and safety than results from standard pairwise analyses,^28^, ^29^ and can rank interventions to inform clinical decisions.^30^ We examined the symmetry and geometry of the data by producing a network plot with node sizes corresponding to number of study participants, and connection sizes corresponding to number of studies for each treatment. We also generated comparison‐adjusted funnel plots to evaluate publication bias and small study effects for all available treatment comparisons,^31^ using Stata V.16 (StataCorp), where there were sufficient studies (≥10 studies).^31^ These are scatterplots of effect size versus precision, measured via the inverse of the standard error. Symmetry around the effect estimate line indicates the absence of publication bias or small‐study effects.^32^ For each treatment, we generated a pooled relative risk (RR) with 95% confidence intervals (CIs) to summarize the effect of each comparison tested using a random effects model as a conservative estimate. We used the RR of failure to achieve complete relief of gastro‐esophageal reflux symptoms, or adequate relief of gastro‐esophageal reflux symptoms, at specific timepoints as the measure of treatment efficacy, whereby if the RR is less than 1 and the 95% CI does not cross 1, there is a significant benefit of one treatment over another, or over placebo. This approach is more stable, compared with RR of improvement, or using the OR, for some meta‐analyses.^33^

We assessed global statistical heterogeneity across all comparisons using the I^2^ measure with the netmeta statistical package. The I^2^ measure ranges from 0% to 100% with a value of 25% to 49% indicating low study heterogeneity, 50% to 74% moderate heterogeneity, and ≥ 75% high heterogeneity.^34^ Moreover, we ranked treatments according to the *P*‐score, which is a value between 0 and 1. *P*‐scores are based solely on the point estimates and standard errors of the network estimates, and measure the extent of certainty that one treatment is better than another, according to any given endpoint, as an average over all other competing treatments.^35^ The higher the *P*‐score, the greater the probability of the treatment being ranked as best,^35^ but magnitude of the *P*‐score should also be considered. As the mean value is always 0.5, if individual treatments cluster around this value, they are likely to have similar efficacies. However, when interpreting the results, it is also important to take the RR and corresponding 95% CI for each comparison into account, rather than relying on rankings alone.^36^

## 4 RESULTS

**FIGURE 1:**
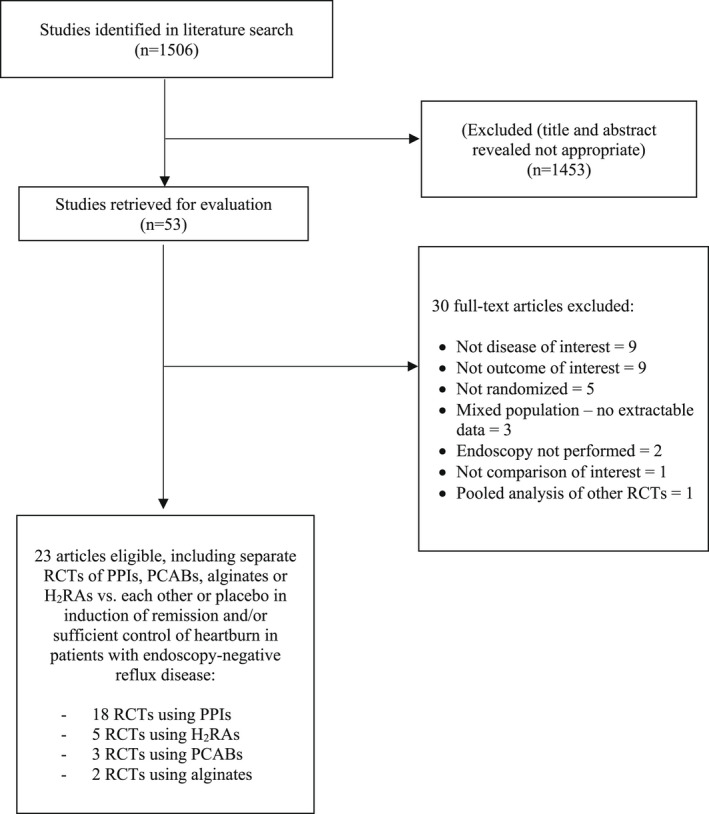
Flow diagram of assessment of studies identified in the network meta‐analysis.

The literature search identified 1506 citations, of which 1453 were excluded on review of the title and abstract (Figure 1). From these, we identified 54 articles appearing relevant to the study question. In total, 23 studies, containing 10,735 subjects with endoscopy‐negative reflux disease, fulfilled all eligibility criteria.^20^, ^21^, ^22^, ^23^, ^37^, ^38^, ^39^, ^40^, ^41^, ^42^, ^43^, ^44^, ^45^, ^46^, ^47^, ^48^, ^49^, ^50^, ^51^, ^52^, ^53^, ^54^, ^55^ Agreement between investigators for assessment of study eligibility was excellent (kappa statistic = 0.85). Overall, 8497 patients received active treatment and 2238 received placebo. Eighteen trials studied the efficacy of active drug versus only placebo,^22^, ^23^, ^39^, ^40^, ^41^, ^42^, ^43^, ^44^, ^45^, ^46^, ^47^, ^48^, ^49^, ^50^, ^51^, ^52^, ^53^, ^54^ 13 of which evaluated PPIs,^39^, ^41^, ^43^, ^44^, ^45^, ^46^, ^47^, ^48^, ^50^, ^51^, ^52^, ^53^, ^54^ three PCABs,^20^, ^21^, ^55^ and two H_2_RAs.^37^, ^38^ Three trials compared efficacy of H_2_RAs with PPIs,^40^, ^42^, ^49^ and two trials alginates versus PPIs.^22^, ^23^ Detailed characteristics of all included studies are provided in Table S1. Patients were allocated to active therapy or placebo as described in Table S2. Risk of bias for all included trials is reported in Table S3; only nine were at low risk of bias across all domains.^20^, ^21^, ^23^, ^46^, ^48^, ^52^, ^53^, ^54^, ^55^

### 4.1 Failure to achieve complete or adequate relief of gastro‐esophageal reflux symptoms

#### 4.1.1 Failure to achieve complete relief of gastro‐esophageal reflux symptoms between ≥2 and <4 weeks of treatment

**FIGURE 2:**
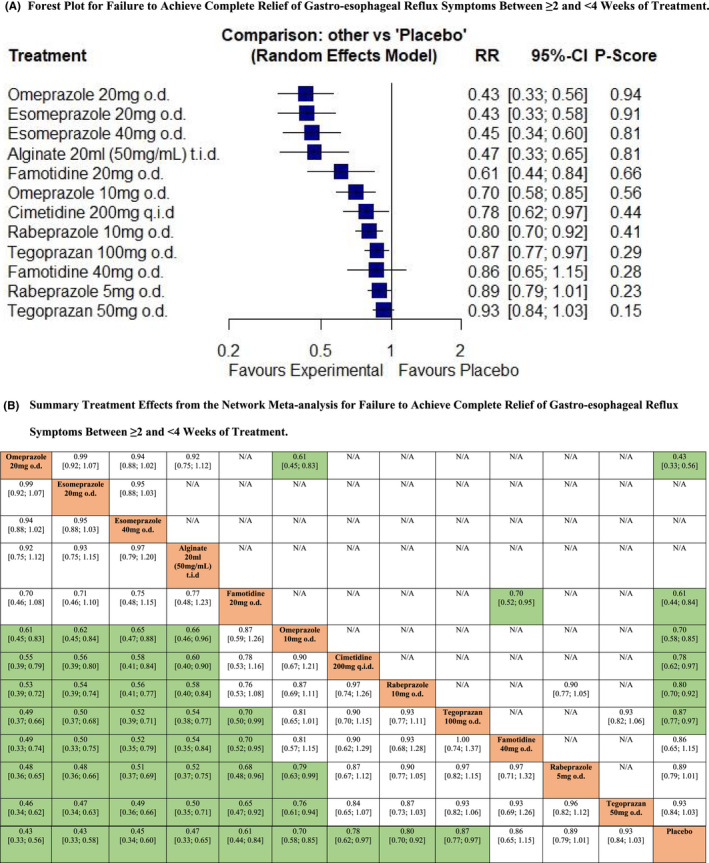
Network meta‐analysis of likelihood of failure to achieve complete relief of gastro‐esophageal reflux symptoms between ≥2 and <4 weeks of treatment. (A) Forest plot for failure to achieve complete relief of gastro‐esophageal reflux symptoms between ≥2 and <4 weeks of treatment. Treatments are reported in order of efficacy ranking according to *P*‐score. The *P*‐score is the probability of each treatment being ranked as best in terms of efficacy in the network. (B) Summary treatment effects from the network meta‐analysis for failure to achieve complete relief of gastro‐esophageal reflux symptoms between ≥2 and <4 weeks of treatment. League table of pairwise comparisons in the network meta‐analysis for the relative risk of failure to achieve complete relief of gastro‐esophageal reflux symptoms between ≥2 and <4 weeks of treatment. Relative risk with 95% confidence intervals in parentheses. Comparisons, column versus row, should be read from left to right, and are ordered relative to their overall efficacy. The treatment in the top left position is ranked as best after the network meta‐analysis of direct and indirect effects. Boxes highlighted in green indicate significant differences. Direct comparisons are provided above the drug labels, and indirect comparisons are below. N/A, not applicable, no RCTs making direct comparisons.

Seven RCTs reported data concerning efficacy of PPIs, H_2_RAs, PCABs, or alginates in terms of failure to achieve complete relief of gastro‐esophageal reflux symptoms between ≥2 and <4 weeks of treatment.^23^, ^37^, ^38^, ^44^, ^47^, ^53^, ^55^ In total, 4325 patients were recruited of whom 3925 received active treatment. The network plot is provided in Figure S1. Pooled analysis revealed no statistical heterogeneity (I^2^ = 0.0%). Omeprazole 20 mg o.d., esomeprazole 20 mg o.d. and 40 mg o.d., 20 ml of alginate t.i.d., famotidine 20 mg o.d., omeprazole 10 mg o.d., cimetidine 200 mg q.i.d., rabeprazole 10 mg o.d. and tegoprazan 100 mg o.d. were all significantly more efficacious than placebo, but omeprazole 20 mg o.d. (RR of failure to achieve complete relief of gastro‐esophageal reflux symptoms = 0.43; 95% CI 0.33 to 0.56, *P*‐score 0.94) ranked first. This means the probability of omeprazole 20 mg o.d. being the most efficacious when all treatments, including placebo, were compared with each other was 94%. However, esomeprazole 20 mg o.d. (RR = 0.43; 95% CI 0.33 to 0.58, *P*‐score 0.91), and 40 mg o.d. (RR = 0.45; 95% CI 0.34 to 0.60, *P*‐score 0.81) performed similarly and were ranked second and third, respectively (Figure 2A). Famotidine 40 mg o.d., rabeprazole 5 mg o.d., and tegoprazan 50 mg o.d. were all no more efficacious than placebo. After indirect comparison of active treatments, omeprazole 20 mg o.d., esomeprazole 20 mg or 40 mg o.d., and 20 ml of alginate t.i.d. were superior to all other active treatments except famotidine 20 mg o.d., which was superior to tegoprazan 100 mg o.d., famotidine 40 mg o.d., rabeprazole 5 mg o.d., and tegoprazan 50 mg o.d. (Figure 2B).

### 4.2 Failure to achieve adequate relief of gastro‐esophageal reflux symptoms between ≥2 and <4 weeks of treatment

**FIGURE 3:**
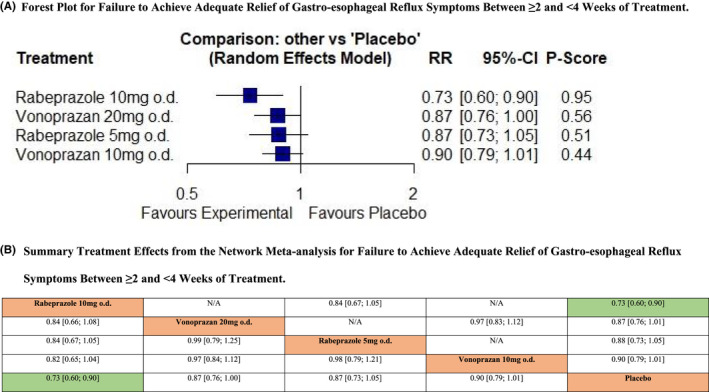
Network meta‐analysis of likelihood of failure to achieve adequate relief of gastro‐esophageal reflux symptoms between ≥2 and <4 weeks of treatment. (A) Forest plot for failure to achieve adequate relief of gastro‐esophageal reflux symptoms between ≥2 and <4 weeks of treatment. Treatments are reported in order of efficacy ranking according to *P*‐score. The *P*‐score is the probability of each treatment being ranked as best in terms of efficacy in the network. (B) Summary treatment effects from the network meta‐analysis for failure to achieve adequate relief of gastro‐esophageal reflux symptoms between ≥2 and <4 weeks of treatment. League table of pairwise comparisons in the network meta‐analysis for the relative risk of failure to achieve adequate relief of gastro‐esophageal reflux symptoms between ≥2 and <4 weeks of treatment. Relative risk with 95% confidence intervals in parentheses. Comparisons, column versus row, should be read from left to right, and are ordered relative to their overall efficacy. The treatment in the top left position is ranked as best after the network meta‐analysis of direct and indirect effects. Boxes highlighted in green indicate significant differences. Direct comparisons are provided above the drug labels, and indirect comparisons are below. N/A, not applicable, no RCTs making direct comparisons

Only three RCTs reported data concerning efficacy of PPIs, H_2_RAs, or PCABs in terms of failure to achieve adequate relief of gastro‐esophageal reflux symptoms between ≥2 and <4 weeks of treatment.^20^, ^21^, ^53^ There were no trials of alginates. In total, 1229 patients were recruited of whom 984 were randomized to active treatment. The network plot is provided in Figure S2. Pooled analysis revealed no heterogeneity between studies (I^2^ = 0.0%), although with only three trials there would be limited power to detect this. Only rabeprazole 10 mg o.d. was significantly more efficacious than placebo, ranking first in the network (RR of failure to achieve adequate relief of gastro‐esophageal reflux symptoms = 0.73; 95% CI 0.60 to 0.90, *P*‐score 0.95) (Figure 3A). Vonoprazan 20 mg or 10 mg o.d., and rabeprazole 5 mg o.d., were all no more efficacious than placebo. After indirect comparison of active treatments, rabeprazole 10 mg o.d. was the only treatment superior to placebo (Figure 3B), but no significant differences were detected with any of the other active treatments.

### 4.3 Failure to achieve complete relief of gastro‐esophageal reflux symptoms at ≥4 weeks of treatment

**FIGURE 4:**
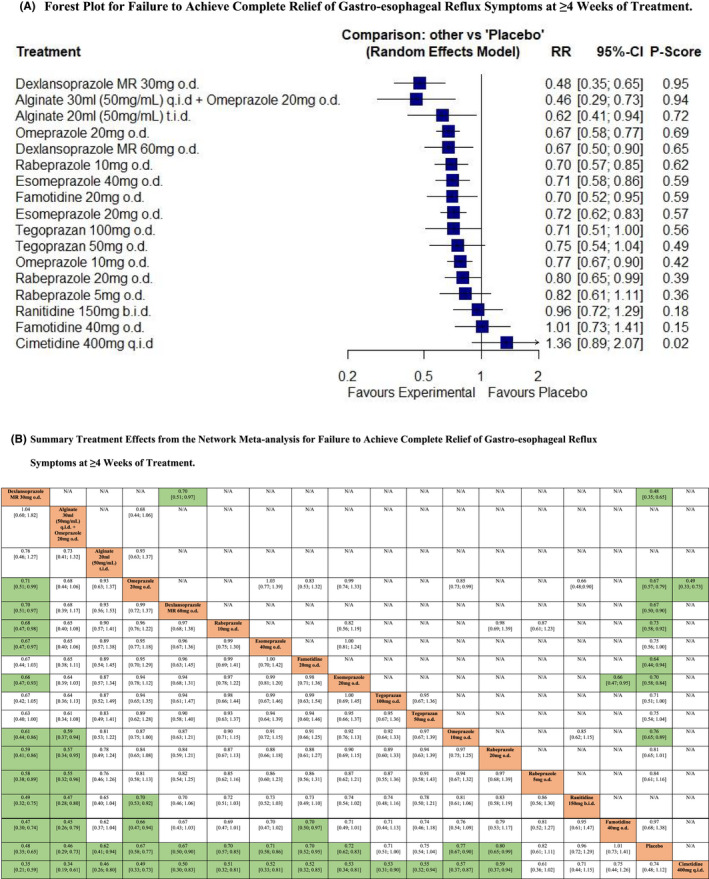
Network meta‐analysis of likelihood of failure to achieve complete relief of gastro‐esophageal reflux symptoms at ≥4 weeks of treatment. (A) Forest plot for failure to achieve complete relief of gastro‐esophageal reflux symptoms at ≥4 weeks of treatment. Treatments are reported in order of efficacy ranking according to *P*‐score. The *P*‐score is the probability of each treatment being ranked as best in terms of efficacy in the network. (B) Summary treatment effects from the network meta‐analysis for failure to achieve complete relief of gastro‐esophageal reflux symptoms at ≥4 weeks of treatment. League table of pairwise comparisons in the network meta‐analysis for the relative risk of failure to achieve complete relief of gastro‐esophageal reflux symptoms at ≥4 weeks of treatment. Relative risk with 95% confidence intervals in parentheses. Comparisons, column versus row, should be read from left to right, and are ordered relative to their overall efficacy. The treatment in the top left position is ranked as best after the network meta‐analysis of direct and indirect effects. Boxes highlighted in green indicate significant differences. Direct comparisons are provided above the drug labels, and indirect comparisons are below. N/A, not applicable, no RCTs making direct comparisons

Twenty RCTs reported data concerning efficacy of PPIs, PCABs, alginates, or H_2_RAs in terms of failure to achieve complete relief of gastro‐esophageal reflux symptoms at ≥4 weeks of treatment.^22^, ^23^, ^38^, ^39^, ^40^, ^41^, ^42^, ^43^, ^44^, ^45^, ^46^, ^47^, ^48^, ^49^, ^50^, ^51^, ^52^, ^53^, ^54^, ^55^ In total, 9287 patients were recruited of whom 7640 were randomized to active treatment. The network plot is provided in Figure S3A. Pooled analysis revealed high levels of statistical heterogeneity (*I* ^2^ = 74.3%), with evidence of funnel plot asymmetry, suggesting publication bias, or other small study effects (Figure S3B). Dexlansoprazole 30 mg o.d. ranked first (RR of failure to achieve complete relief of gastro‐esophageal reflux symptoms = 0.48, 95% CI 0.35 to 0.65, *P*‐score 0.95), with 30 ml of alginate q.i.d. in combination with omeprazole 20 mg o.d. performing similarly and ranked second (RR = 0.46, 95% CI 0.29 to 0.73, *P*‐score 0.94), and 30 ml of alginate t.i.d. ranked third (RR = 0.62, 95% CI 0.41 to 0.94, *P*‐score 0.72) (Figure 4A). However, for the latter two, these results were based on one trial recruiting 76 patients, and one trial in 195 patients.^22^, ^23^ Tegoprazan 100 mg o.d. and 50 mg o.d., rabeprazole 5 mg o.d., ranitidine 150 mg b.i.d., famotidine 40 mg o.d., and cimetidine 400 mg q.i.d. were all no more efficacious than placebo. After indirect comparison of active treatments, dexlansoprazole 30 mg o.d. was superior to omeprazole 20 mg and 10 mg o.d., dexlansoprazole 60 mg o.d., rabeprazole 20 mg, 10 mg, or 5 mg o.d., esomeprazole 40 mg or 20 mg o.d., ranitidine 150 mg b.i.d., famotidine 40 mg o.d., cimetidine 400 mg q.i.d., and placebo (Figure 4B).

### 4.4 Failure to achieve adequate relief of gastro‐esophageal reflux symptoms at ≥4 weeks of treatment

**FIGURE 5:**
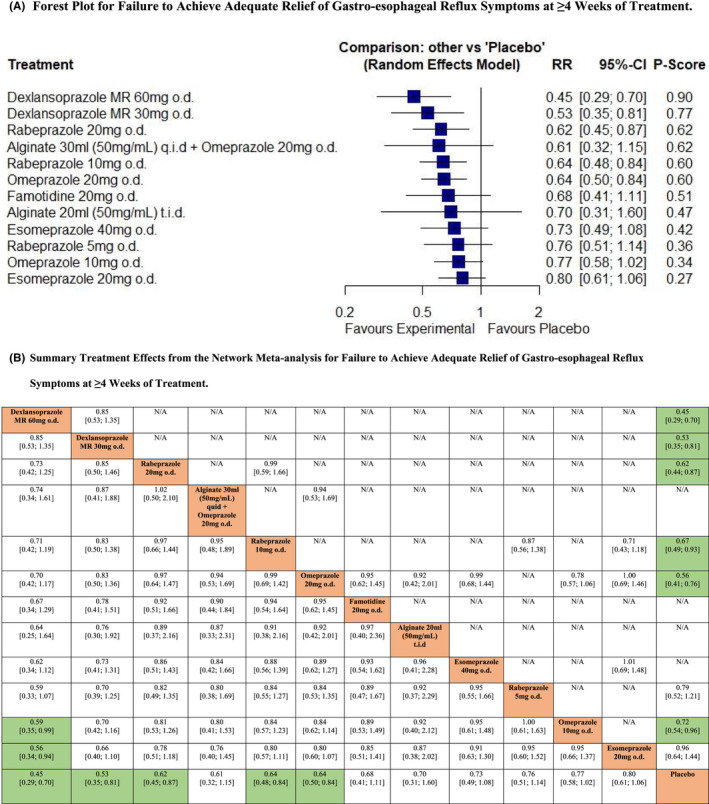
Network meta‐analysis of likelihood of failure to achieve adequate relief of gastro‐esophageal reflux symptoms at ≥4 weeks of treatment. (A) Forest plot for failure to achieve adequate relief of gastro‐esophageal reflux symptoms at ≥4 weeks of treatment. Treatments are reported in order of efficacy ranking according to *P*‐score. The *P*‐score is the probability of each treatment being ranked as best in terms of efficacy in the network. (B) Summary treatment effects from the network meta‐analysis for failure to achieve adequate relief of gastro‐esophageal reflux symptoms at ≥4 weeks of treatment. League table of pairwise comparisons in the network meta‐analysis for the relative risk of failure to achieve adequate relief of gastro‐esophageal reflux symptoms at ≥4 weeks of treatment. Relative risk with 95% confidence intervals in parentheses. Comparisons, column versus row, should be read from left to right, and are ordered relative to their overall efficacy. The treatment in the top left position is ranked as best after the network meta‐analysis of direct and indirect effects. Boxes highlighted in green indicate significant differences. Direct comparisons are provided above the drug labels, and indirect comparisons are below. N/A, not applicable, no RCTs making direct comparisons.

Twelve RCTs reported data concerning efficacy of all the interventions of interest in terms of failure to achieve adequate relief of gastro‐esophageal reflux symptoms at ≥4 weeks of treatment.^22^, ^23^, ^41^, ^43^, ^45^, ^47^, ^48^, ^49^, ^50^, ^52^, ^53^, ^54^ In total, 5793 patients were recruited of whom 4898 received active treatment. The network plot is provided in Figure S4A. Pooled analysis revealed moderate levels of statistical heterogeneity (*I* ^2^ = 67.2%). There was no evidence of funnel plot asymmetry, suggesting publication bias, or other small study effects (Figure S4B). Dexlansoprazole 60 mg o.d. ranked first (RR of failure to achieve adequate relief of gastro‐esophageal reflux symptoms = 0.45; 95% CI 0.29 to 0.70, *P*‐score 0.90), dexlansoprazole 30 mg o.d. second (RR = 0.53; 95% CI 0.35 to 0.81, *P*‐score 0.77), and rabeprazole 20 mg o.d. third (RR = 0.62; 95% CI 0.45 to 0.87, *P*‐score 0.62) (Figure 5A). Rabeprazole 10 mg o.d. and omeprazole 20 mg o.d. were also significantly more efficacious than placebo. 30 ml of alginate q.i.d. in combination with omeprazole 20 mg o.d., famotidine 20 mg o.d., 20 ml of alginate t.i.d., esomeprazole 40 mg or 20 mg o.d., rabeprazole 5 mg o.d., and omeprazole 10 mg o.d. were all no more efficacious than placebo. After indirect comparison of active treatments, dexlansoprazole 60 mg o.d. was superior to omeprazole 10 mg o.d. and esomeprazole 20 mg o.d. but there were no other significant differences (Figure 5B).

### 4.5 Adverse events

Total numbers of adverse events were reported by 17 RCTs containing 6598 patients.^20^, ^21^, ^22^, ^23^, ^37^, ^38^, ^39^, ^40^, ^42^, ^44^, ^45^, ^46^, ^48^, ^51^, ^52^, ^53^, ^55^ There were 4827 patients randomized to active treatment. The network plot is provided in Figure S5A. Pooled analysis revealed minimal statistical heterogeneity (*I* ^2^ = 10.9%) and no evidence of funnel plot asymmetry (Figure S5B). None of the active treatments were more likely to lead to adverse events, compared with placebo (Figure S5C), but cimetidine 400 mg q.i.d. ranked first for safety (*P*‐score 0.85). On indirect comparison, cimetidine 400 mg q.i.d. was significantly less likely to lead to adverse events than either omeprazole 20 mg o.d. or ranitidine 150 mg q.i.d.

There were 17 RCTs that provided adverse events leading to withdrawal of therapy recruiting 6797 patients, 5080 of whom were randomized to active treatment.^20^, ^21^, ^22^, ^23^, ^37^, ^38^, ^39^, ^40^, ^41^, ^42^, ^44^, ^46^, ^48^, ^51^, ^52^, ^54^, ^55^ The network plot is provided in Figure S6A. When data were pooled, there was no statistical heterogeneity (*I* ^2^ = 0.0%) and no evidence of funnel plot asymmetry (Figure S6B). None of the active treatments were more likely to lead to withdrawal due to adverse events, compared with placebo (Figure S6C), with omeprazole 10 mg o.d. ranked first (*P*‐score 0.78). On indirect comparison, omeprazole 10 mg o.d. was significantly less likely to lead to withdrawals due to adverse events than vonoprazan 10 mg o.d.

## 5 DISCUSSION

In this systematic review and network meta‐analysis, we compared the efficacy of PPIs, H_2_RAs, PCABs, and alginates, versus each other, or placebo, in endoscopy‐negative reflux disease. In terms of failure to achieve complete relief of gastro‐esophageal reflux symptoms between ≥2 and <4 weeks of treatment, omeprazole 20 mg o.d. appeared to be the best treatment, although esomeprazole 20 mg o.d. and 40 mg o.d. performed similarly and were ranked second and third, respectively. In terms of failure to achieve adequate relief of symptoms between ≥2 and <4 weeks of treatment, only rabeprazole 10 mg o.d. was significantly more efficacious than placebo. For failure to achieve complete relief of symptoms ≥4 weeks of treatment, dexlansoprazole 30 mg o.d. ranked first, with 30 ml of alginate q.i.d. in combination with omeprazole 20 mg o.d. performing similarly and ranked second, and 30 ml of alginate t.i.d. ranked third. Regarding failure to achieve adequate relief of symptoms after ≥4 weeks of treatment, dexlansoprazole 60 mg o.d. ranked first, with dexlansoprazole 30 mg o.d. second, and rabeprazole 20 mg o.d. third. All drugs were safe and well tolerated, but cimetidine 400 mg q.i.d. ranked first for safety, in terms of overall adverse events. Finally, none of the drugs were more likely to lead to withdrawal due to adverse events, compared with placebo, with omeprazole 10 mg o.d. ranked first.

We used standard methodology to maximize the likelihood of identifying all pertinent literature and minimize bias. The literature search, eligibility assessment, and data extraction for this network meta‐analysis were undertaken independently by two reviewers, with any discrepancies resolved by consensus. We used an intention‐to‐treat analysis, with all dropouts assumed to have failed therapy, and pooled data with a random effects model, to reduce the likelihood that any beneficial effect of PPIs, H_2_RAs, PCABs, or alginates has been overestimated. Limitations of this study include the fact that our conclusions are limited by the quality of the eligible included trials. Only nine were low risk of bias across all domains. Therefore, the results of the network meta‐analysis should be interpreted with caution. Trials that do not report their methodology in sufficient detail tend to overestimate the efficacy of the active intervention studied. Moreover, a wide range of measures of treatment efficacy were used and were reported at various timepoints in the studies. This is particularly pertinent with regard to the definition of adequate relief, with trials using various and different criteria to assess this and, often, it was dependent on subjective evaluation. Therefore, with these considerations in mind, the network's findings on adequate relief may need to be interpreted with caution. However, we standardized this as much as possible in our analyses, according to the criteria used to define complete or adequate relief of gastro‐esophageal reflux symptoms. In addition, particularly, when we evaluated the efficacy of treatments in achieving complete relief of gastro‐esophageal reflux symptoms at ≥4 weeks, for 30 ml of alginate q.i.d. in combination with omeprazole 20 mg o.d. and 30 ml of alginate t.i.d. only one relatively small trial contributed to the outcome, meaning that these results should be interpreted cautiously. One final criticism that could be leveled at this study is most included studies defined patients as having endoscopy‐negative reflux disease without performing pH‐monitoring studies, on the basis of typical symptoms and an endoscopy that confirmed no evidence of EE. This means that some of the trials probably included patients with reflux hypersensitivity or functional heartburn, which may be less likely to respond to acid suppressant drugs or alginates.^13^, ^56^, ^57^ This may have contributed to the lack of a dose–response effect seen with PPIs, with omeprazole 20 mg o.d. ranking first between ≥2 and <4 weeks of treatment, despite its lower antisecretory capability and high interindividual variability in effect.^58^

A previous network meta‐analysis, including 15 trials published before 2011, investigated the efficacy and safety of PPIs in treating endoscopy‐negative reflux disease.^59^ The authors reported that omeprazole 20 mg o.d. achieved the highest rate of symptomatic relief compared with omeprazole 10 mg o.d. or rabeprazole 5 mg o.d., and that dexlansoprazole 30 mg o.d. therapy significantly improved the rate of symptomatic relief compared with rabeprazole 5 mg o.d. However, in terms of symptomatic relief, there was no distinction between complete and adequate relief. Additionally, the duration of included studies was variable, and efficacy according to treatment duration was not studied. In terms of safety, the authors reported no significant difference between drugs, although included studies had variable follow‐up of between 1 and 6 months. Another meta‐analysis found that PPIs were significantly superior to H_2_RAs and placebo for the symptomatic relief of endoscopy‐negative reflux disease.^60^ Although rates of symptomatic relief according to treatment duration were studied, again there was no distinction between complete and adequate relief of symptoms. PPIs may be superior to H_2_RAs due their more profound acid suppressive effects. H_2_RAs only inhibit the acid secretion stimulated by gastrin partially and are more effective for inhibiting intra‐gastric acidity during periods of basal acid secretion, such as at night.^61^ However, although evening dosing regimens provide prolonged nocturnal acid suppression, they are not as effective at increasing daytime intra‐gastric pH and cannot overcome food‐stimulated acid secretion.^62^ Furthermore, H_2_RAs are not effective for suppressing pepsin secretion during the daytime, as shown in many 24 hour pH‐monitoring studies.^63^, ^64^

Another systematic review with meta‐analysis evaluated the efficacy of PPIs (10 trials), H_2_RAs (five trials) and prokinetics (one trial) for both complete and adequate heartburn relief in patients with endoscopy‐negative reflux disease.^19^ Similar to our results, the authors concluded that PPIs were more efficacious than H_2_RAs, in terms of complete and adequate heartburn relief. Moreover, they demonstrated that PPIs were more efficacious than cisapride. As international guidelines do not recommend prokinetics as monotherapy or add‐on therapy for routine GERD management,^17^, ^65^ we did not include trials on prokinetics in our network. Most of these prior meta‐analyses included patients with endoscopy‐negative reflux disease diagnosed according to a negative upper endoscopy and typical GERD symptoms.

Our network meta‐analysis provides updated evidence on PPIs and H_2_RAs for endoscopy‐negative reflux disease, as well as data for PCABs and alginates, whose efficacy and safety in endoscopy‐negative reflux disease had not been examined in previous meta‐analyses. Regarding PCABs, to date, only tegoprazan has been approved for endoscopy‐negative reflux disease in South Korea.^66^ Four weeks of tegoprazan 50 mg and 100 mg have been demonstrated to be more efficacious than placebo in achieving complete resolution of both heartburn and regurgitation.^55^ Conversely, vonoprazan showed variable results in this patient group.^20^, ^21^ Our results, although based on only three trials, demonstrated that tegoprazan 100 mg o.d. was only just superior to placebo for achieving complete relief between ≥2 and <4 weeks of treatment. All other results for PCABs failed to reach statistical significance. This does not appear to be the case for patients with EE, where a recent network meta‐analysis demonstrated that vonoprazan 20 mg o.d. was at least as effective as PPIs, in terms of heartburn resolution at Day 1 and Day 7.^67^ Therefore, we believe further RCTs of PCABs are necessary in patients with endoscopy‐negative reflux disease to confirm their efficacy.

Controlling heartburn in patients with endoscopy‐negative reflux disease can also be achieved with alginate compounds, characterized by a unique mechanism of action with the mechanical formation of a raft floating above gastric secretions.^68^ In fact, trials have demonstrated their benefit in both patients with endoscopy‐negative reflux disease and EE, both as monotherapy and add‐on therapy to PPIs.^22^, ^25^ In the trial by Manabe et al.,^22^ included in our network, patients who received omeprazole combined with sodium alginate reported longer symptom relief compared with those receiving omeprazole alone. The authors concluded that sodium alginate should be considered for treating endoscopy‐negative reflux disease patients who do not respond completely to PPIs. Another prospective study investigated the role of sodium alginate as monotherapy both in patients with endoscopy‐negative reflux disease and EE, finding similar effects on symptom relief in these two groups.^25^ Our network showed that, in terms of achieving complete relief of symptoms between ≥2 and <4 weeks of treatment, 20 ml of alginate t.i.d. ranked fourth and performed similarly to omeprazole 20 mg o.d. and esomeprazole 20 mg or 40 mg o.d. In addition, 30 ml of alginate q.i.d. combined with omeprazole 20 mg and 20 ml of alginate t.i.d. ranked second and third in terms of achieving complete relief of symptoms at ≥4 weeks of treatment. However, only a single study of alginates, containing relatively few patients, contributed to these analyses, and further RCTs are needed to confirm their efficacy in endoscopy‐negative reflux disease. Additionally, available alginates have different compositions, with a wide spectrum of alginate‐based material. As a consequence, the in vitro and in vivo behavior of each formulation may be different, and results obtained with one product should not be extrapolated to others.^69^ None of the RCTs we identified used Gaviscon, perhaps the most widely recognized.

Although endoscopy‐negative reflux disease represents the most common phenotypic presentation of GERD, less is known about the efficacy of the different available drugs in patients with this condition compared with EE. In addition, in the last 15 years, it has become clear that patients with endoscopy‐negative reflux disease are markedly heterogeneous from a pathological and clinical point of view, and should be further subclassified by means of pH‐impedance testing.^14^ This technique is able to detect any kind of chemical reflux and has enabled such patients to be divided into several subgroups on the basis of their reflux patterns, including those with reflux hypersensitivity or functional heartburn, whose symptoms may be less responsive to acid‐suppressive treatments.^13^, ^14^, ^57^ This should be incorporated into the design of future therapeutic trials in patients with endoscopy‐negative reflux disease. Despite the limitations, as discussed, which relate to the included and available trials, we believe our network meta‐analysis is the first to report efficacy and safety of PPIs, H_2_RAs, PCABs, and alginates compared with each other or with placebo in patients with endoscopy‐negative reflux disease. Our results confirm the superiority of PPIs, as a class, compared with other drugs in treating endoscopy‐negative reflux disease. However, it must be noted that generic formulations of PPIs may have reduced drug absorption and efficacy due to intragastric degradation of the active ingredient, compared with branded PPI formulations.^70^ Nevertheless, the majority of RCTs did not report whether they used generic or branded PPIs.

In summary, in the short‐term, omeprazole 20 mg o.d. and esomeprazole 20 mg o.d. or 40 mg o.d. were the best treatments in terms of achieving complete relief of symptoms, whereas rabeprazole 10 mg o.d. performed best for adequate relief. In the longer term, dexlansoprazole 30 mg o.d., 30 ml of alginate q.i.d. combined with omeprazole 20 mg o.d., and 30 ml of alginate t.i.d. were ranked first, second, and third in achieving complete relief of symptoms, although the latter two each based on data from one relatively small RCT. Dexlansoprazole 60 mg or 30 mg o.d. and rabeprazole 20 mg o.d. were the most efficacious for adequate relief. Our results question the efficacy of most PCABs, other than tegoprazan 100 mg o.d., for endoscopy‐negative reflux disease. Future RCTs should aim to better classify patients with endoscopy‐negative reflux disease, and to establish the role of alginates and PCABs in achieving adequate and complete symptoms relief in endoscopy‐negative reflux disease in both the short and long‐term.

## AUTHOR CONTRIBUTIONS

BB, PV, EVS, NdB, CJB, and ACF and conceived and drafted the study. BB and PV collected all data. BB, PV, EVS, CJB, and ACF interpreted all data. BB, CJB, and ACF analyzed all data. BB, PV, EVS, CJB, and ACF drafted the manuscript. All authors commented on drafts of the paper. All authors have approved the final draft of the manuscript.

## CONFLICT OF INTEREST

Brigida Barberio: None. Pierfrancesco Visaggi: None. Edoardo V Savarino: None. Nicola de Bortoli: None. Christopher J. Black: None. Alexander C. Ford: None. Guarantor of the article: EVS is guarantor.

## Supporting information


Data S1

## Data Availability

The data that supports the findings of this study are available in the supplementary material of this article
