# Supplementary material for: Comparison of acid‐lowering drugs for endoscopy negative reflux disease: Systematic review and network Meta‐Analysis
Source: Neurogastroenterol Motil. 2022 Sep 25;35(1):e14469. doi: 10.1111/nmo.14469 (PMC10078414; doi:10.1111/nmo.14469)
Supplement: Supplementary file 1 — Data S1 [file NMO-35-0-s001.docx]

**SUPPLEMENTARY TABLES AND FIGURES**

**Supplementary Table 1. Characteristics of Included Studies of Randomized Controlled Trials of PPIs, H_2_RAs, PCABs or Alginates in Patients with Non-erosive Reflux Disease.**

| **Study and year** | **Country** | **Number of centers** | **Treatments compared (No. of patients)** | **Type and name of Drug(s)** | **Duration of active treatment** | **Symptoms assessed** | **Endpoint(s)**  **(relief of gastro-esophageal reflux)** | **Definition of NERD** |
| --- | --- | --- | --- | --- | --- | --- | --- | --- |
| **Riemann 1991**^1^ | Germany | Not specified | a) Cimetidine 200mg q.i.d. (60)  b) Placebo (65) | Not reported | 2 weeks | Heartburn and/or regurgitation | Complete relief | Heartburn and regurgitation of acid (self-assessment of symptoms by patient) daily for at least 5 days in the pre-trial week, and endoscopy within 5 days before  study entry revealing no erosive esophageal changes |
| **Robinson 1991**^2^ | United States | 22 | a) Famotidine 40mg o.d. (155)  b) Famotidine 20mg b.i.d. (158)  c) Placebo (76) | Not reported | 2 weeks | Heartburn | Adequate relief (moderate or excellent improvement according to patient opinion at week 2 compared with baseline) | Heartburn, characterized by  retrosternal burning pain present for approximately 15 out of 30 days prior to  entry and endoscopic evidence of a lack of any break in the  esophageal mucosa |
| **Bate 1996^3^** | Ireland, United Kingdom | 23 | a) Omeprazole 20mg o.d. (98)  b) Placebo (111) | Not reported | 4 weeks | Heartburn and/or regurgitation | Complete relief | Heartburn with or without regurgitation and normal esophageal mucosa verified by endoscopy no more than 3 days prior to randomization |
| **Bate 1997**^4^ | United Kingdom | 19 | a) Cimetidine 400mg q.i.d. (109)  b) Omeprazole 20mg o.d. (112) | a) Generic (Generics Ltd, Potters Bar, UK)  b) Branded  (Losec; Astra Pharmaceuticals Ltd, Kings Langley, UK) | 4 weeks | Heartburn | Complete relief | Heartburn with or without regurgitation and normal esophageal mucosa verified by endoscopy |
| **Lind 1997^5^** | Denmark, Sweden | 25 | a) Omeprazole 20mg o.d. (205)  b) Omeprazole 10mg o.d. (199)  c) Placebo (105) | Not reported | 4 weeks | Heartburn | Complete relief Adequate relief (≤1 day with mild episodes of heartburn during the past 1 week at week 4) | History of  heartburn as the predominant symptom during the past 12  months, with episodes of heartburn occurring on at least 2  days weekly, and without any endoscopic signs of esophagitis |
| **Venables 1997^6^** | United Kingdom | 106 | a) Ranitidine 150mg b.i.d. (326)  b) Omeprazole 20mg o.d. (330)  c) Omeprazole 10mg o.d. (338) | Not reported | 4 weeks | Heartburn | Complete relief | Heartburn as the predominant symptom of  GERD for at least the previous 3 months and absence of erosive esophagitis at randomization |
| **Carlsson 1998^7^** | Australia, Holland, Norway, United Kingdom | 36 | a) Omeprazole 20mg o.d. (87)  b) Omeprazole 10mg o.d. (86)  c) Placebo (88) | Not reported | 4 weeks | Heartburn and/or regurgitation | Complete relief Adequate relief (not specified) | History of upper gastrointestinal symptoms for at least 3 months occurring at least 2 days during the last 7 days prior to endoscopy and absence of endoscopic mucosal breaks |
| **Richter 2000^8^** | United States | 36 | a) Omeprazole 20mg o.d. (118)  b) Omeprazole 10mg o.d. (118)  c) Placebo (123) | Not reported | 4 weeks | Heartburn | Complete relief | Heartburn as the predominant symptom for at least 3 months and absence of erosive esophagitis. |
| **Miner 2002^9^** | United States | 19 | a) Rabeprazole 20mg o.d. (68)  b) Rabeprazole 10mg o.d. (65)  c) Placebo (70) | Not reported | 4 weeks | Heartburn and/or regurgitation | Complete relief  Adequate relief (≤1 episode of moderate severity during the preceding 1 week at week 4) | Heartburn with or without regurgitation for at least 3 months and no macroscopic erosion on endoscopy. |
| **Katz 2003^10^** | United States | 53 | a) Esomeprazole 40mg o.d. (241)  b) Esomeprazole 20mg o.d. (234)  c) Placebo (242) | Not reported | 4 weeks | Heartburn | Complete relief | History of heartburn for at least the previous 6 months and no evidence of erosive esophagitis. |
| **Armstrong 2004^11^** | Australia,Canada,Denmark, Finland,  France, Germany, Norway, Sweden, United Kingdom, United States | Not specified | a) Esomeprazole 40mg o.d. (772)  b) Esomeprazole 20mg o.d. (759)  c) Omeprazole 20mg o.d. (1114) | Not reported | 4 weeks | Heartburn | Complete relief  Adequate relief (≤1 day of mild heartburn during the last 1 week before the last visit) | Heartburn as predominant  symptom for 6 months or longer, and for four days or more  during the last week before the start of the study and normal endoscopy. |
| **Fock 2005^12^** | Singapore | Not specified | a) Esomeprazole 20mg o.d. (64)  b) Rabeprazole 10mg o.d. (63) | Not reported | 4 weeks | Heartburn and/or regurgitation | Complete relief  Adequate relief (no episodes of moderate or severe symptoms during week 4) | Heartburn or regurgitation in the last 7 days prior to randomization and no esophageal mucosal break on endoscopy. |
| **Fujiwara 2005^13^** | Japan | 8 | a) Famotidine 20mg o.d. (53)  b) Omeprazole 20mg o.d. (53) | Not reported | 4 weeks | Heartburn and/or regurgitation | Complete relief  Adequate relief (decrease in frequency of symptoms during a 7-day interval in week 4 compared with baseline) | Heartburn and/or regurgitation at least twice weekly for ≥1 month and no mucosal break on endoscopy. |
| **Kahrilas 2005^14^** | United States | 38 | a) Rabeprazole 20mg o.d. (129)  b) Placebo (132) | Not reported | 4 weeks | Heartburn | Complete relief  Adequate relief (symptom score of 0 (no symptoms) or 1 (slight symptoms) for a 24-hour period at day 28 | GERD symptoms without erosive esophagitis on endoscopy. |
| **Uemura 2008^15^** | Japan | 33 | a) Omeprazole 20mg o.d. (93)  b) Omeprazole 10mg o.d. (96)  c) Placebo (95) | Not reported | 4 weeks | Heartburn | Complete relief  Adequate relief (no heartburn or ≤1 day with mild heartburn for 7 consecutive days during week 4) | Heartburn as predominant symptoms for at least one month before screening without erosive esophagitis on endoscopy |
| **Fass 2009^16^** | United States | 153 | a) Dexlansoprazole 60mg MR o.d. (315)  b) Dexlansoprazole 30mg MR o.d. (315)  c) Placebo (317) | Branded  (TAK-390MR; Takeda Global Research & Development Center, Inc., Deerfield, IL, USA) | 4 weeks | Heartburn | Complete relief  Adequate relief (improvement in heartburn severity on a five-point scale at week 4) | History of heartburn for 6 months or longer, heartburn on at least 4 of the 7 days preceding randomization and normal esophageal mucosa at the screening endoscopy. |
| **Kinoshita 2011^17^** | Japan | 35 | a) Rabeprazole 10mg o.d. (102)  b) Rabeprazole 5mg o.d. (93)  c) Placebo (93) | Not reported | 4 weeks | Heartburn | Complete relief  Adequate relief (≤1 period (daytime or night-time) of heartburn during seven days before week 4 | History of heartburn for at least 2 days a week for 3 consecutive weeks before screening visit and absence of erosive esophagitis on endoscopy. |
| **Tan 2011^18^** | China | 1 | a) Esomeprazole 20mg o.d. (85)  b) Placebo (90) | Not reported | 8 weeks | Heartburn and/or regurgitation | Complete relief  Adequate relief (mild, acceptable symptoms according to patient opinion) | Reflux-related symptoms in the  absence of mucosal erosions and/or breaks on endoscopy. |
| **Manabe 2012^19^** | Japan | 11 | a) Alginate 30 ml (50mg/ml) q.i.d. and Omeprazole 20mg o.d. (36)  b) Omeprazole 20mg o.d. (40) | a) Branded (Alloid G; Kaigen Co. Ltd, Osaka, Japan)  b) Not reported | 4 weeks | Heartburn | Complete relief  Adequate relief (complete  resolution or only mild symptoms on 1 day per week at end of treatment) | History of heartburn for at least 2 days a week for one month before screening and absence of esophageal mucosal breaks on endoscopy. |
| **Chiu 2013^20^** | Taiwan | 1 | a) Sodium alginate 1000mg t.i.d. (97)  b) Omeprazole 20mg o.d. (98) | a) Branded (Alginos; Center Laboratories, Inc., Hsinchu, Taiwan)  b) Branded (Omelon; YSP Ind. Co., Ltd., Taichung, Taiwan) | 4 weeks | Heartburn and/or regurgitation | Complete relief  Adequate relief (≤1 day with mild heartburn  or regurgitation episodes in the last 1 week at week 4) | History of heartburn for at least 2 days a week for one month before screening and absence of esophageal mucosal breaks on endoscopy. |
| **Kinoshita 2016^21^** | Japan | 75 | a) Vonoprazan 20mg o.d. (271)  b) Vonoprazan 10mg o.d. (278)  c) Placebo (278) | Not reported | 2 weeks | Heartburn | Adequate relief (proportion of days without heartburn higher compared with run-in period) | History of heartburn for at least 2 days a week for 3 consecutive weeks before run-in period and absence of erosive esophagitis on endoscopy. |
| **Kinoshita 2019^22^** | Japan | 30 | a) Vonoprazan 10mg o.d. (240)  b) Placebo (245) | Not reported | 2 weeks | Heartburn | Adequate relief (reduction in proportion of days with heartburn at week 2 compared with run-in period) | History of heartburn for at least 2 days a week for 3 consecutive weeks before run-in period and absence of erosive esophagitis on endoscopy. |
| **Kim 2021^23^** | South Korea | 17 | a) Tegoprazan 100mg o.d. (108)  b) Tegoprazan 50mg o.d. (108)  c) Placebo (108) | Not reported | 4 weeks | Heartburn and/or regurgitation | Complete relief | Heartburn and regurgitation for at least 3 months before screening, heartburn and regurgitation for 7 days prior to randomization, and absence of erosive esophagitis on endoscopy. |

**Abbreviations:**

GERD: gastro-esophageal reflux disease; MR: modified release; o.d.: once daily; b.i.d: twice daily; t.i.d.: three times daily; q.i.d.: four times daily

**Supplementary Table 2. Total Number of Trials of Each Treatment, and Total Number of Included Patients Assigned to Each Drug and Placebo in Randomized Controlled Trials of PPIs, H_2_RAs, PCABs or Alginates in Patients with Non-erosive Reflux Disease.**

| **Treatment** | **Number of RCTs** | **Total Number of Patients** | **References** |
| --- | --- | --- | --- |
| **Cimetidine 200mg q.i.d.** | 1 | 60 | 1 |
| **Cimetidine 400mg q.i.d.** | 1 | 109 | 4 |
| **Famotidine 20mg b.i.d.** | 2 | 211 | 2, 13 |
| **Famotidine 40mg o.d.** | 1 | 155 | 2 |
| **Ranitidine 150mg b.i.d** | 1 | 326 | 6 |
| **Dexlansoprazole 30mg o.d.** | 1 | 315 | 16 |
| **Dexlansoprazole 60mg o.d.** | 1 | 315 | 16 |
| **Esomeprazole 20mg o.d.** | 4 | 1151 | 10, 11, 12, 18 |
| **Esomeprazole 40mg o.d.** | 2 | 1013 | 10, 11 |
| **Omeprazole 10mg o.d.** | 5 | 836 | 5, 6, 7, 8, 15 |
| **Omeprazole 20mg o.d.** | 11 | 2348 | 3, 4, 5, 6, 7, 8, 11, 13, 15, 19, 20 |
| **Rabeprazole 5mg o.d.** | 1 | 93 | 17 |
| **Rabeprazole 10mg o.d.** | 3 | 230 | 9, 12, 17 |
| **Rabeprazole 20mg o.d.** | 2 | 197 | 9, 14 |
| **Sodium alginate 1000mg t.i.d.** | 1 | 97 | 20 |
| **Alginate 30 ml (50mg/ml) q.i.d. and Omeprazole 20mg o.d.** | 1 | 36 | 19 |
| **Vonoprazan 10mg o.d.** | 2 | 518 | 21, 22 |
| **Vonoprazan 20mg o.d.** | 1 | 271 | 21 |
| **Tegoprazan 50mg o.d.** | 1 | 108 | 23 |
| **Tegoprazan 100mg o.d.** | 1 | 108 | 23 |
| **Placebo** | 16 | 2238 | 1, 2, 3, 5, 7, 8, 9, 10, 14, 15, 16, 17, 18, 21, 22, 23 |

**Supplementary Table 3. Risk of Bias of Randomized Controlled Trials of PPIs, H2RAs, PCABs or Alginates in Patients with Non-erosive Reflux Disease.**

| **Study and year** | **Method of Generation of Randomization Schedule** | **Method of Concealment of Treatment Allocation** | **Blinding** | **Evidence of Incomplete Outcomes Data** | **Evidence of Selective Reporting of Outcomes** |
| --- | --- | --- | --- | --- | --- |
| **Riemann 1991^1^** | Unclear | Low | Unclear | Low | Low |
| **Robinson 1991^2^** | Unclear | Unclear | Unclear | Low | Unclear |
| **Bate 1996^3^** | Unclear | Unclear | Low | Low | Low |
| **Bate 1997^4^** | Unclear | High | Unclear | Low | Low |
| **Lind 1997^5^** | Low | Unclear | Low | Low | Unclear |
| **Venables 1997^6^** | Unclear | Low | Low | Low | Low |
| **Carlsson 1998^7^** | Unclear | Unclear | Low | Low | Low |
| **Richter 2000^8^** | Unclear | Unclear | Unclear | Low | Unclear |
| **Miner 2002^9^** | Unclear | Unclear | Unclear | Low | Unclear |
| **Katz 2003^10^** | Low | Low | Low | Low | Low |
| **Armstrong 2004^11^** | Unclear | Low | Unclear | Low | Low |
| **Fock 2005^12^** | Low | Low | Low | Low | Low |
| **Fujiwara 2005^13^** | Unclear | High | Low | Low | Low |
| **Kahrilas 2005^14^** | Unclear | Unclear | Low | Low | Unclear |
| **Uemura 2008^15^** | Unclear | Unclear | Unclear | Low | Low |
| **Fass 2009^16^** | Low | Low | Low | Low | Low |
| **Kinoshita 2011^17^** | Low | Low | Low | Low | Low |
| **Tan 2011^18^** | Low | Low | Low | Low | Low |
| **Manabe 2012^19^** | Low | High | Low | Low | Unclear |
| **Chiu 2013^20^** | Low | Low | Low | Low | Low |
| **Kinoshita 2016^21^** | Low | Low | Low | Low | Low |
| **Kinoshita 2019^22^** | Low | Low | Low | Low | Low |
| **Kim 2021^23^** | Low | Low | Low | Low | Low |

**Supplementary Figure 1. Network Plot for Failure to Achieve Complete Relief Between ≥2 and <4 Weeks of Treatment.**

**
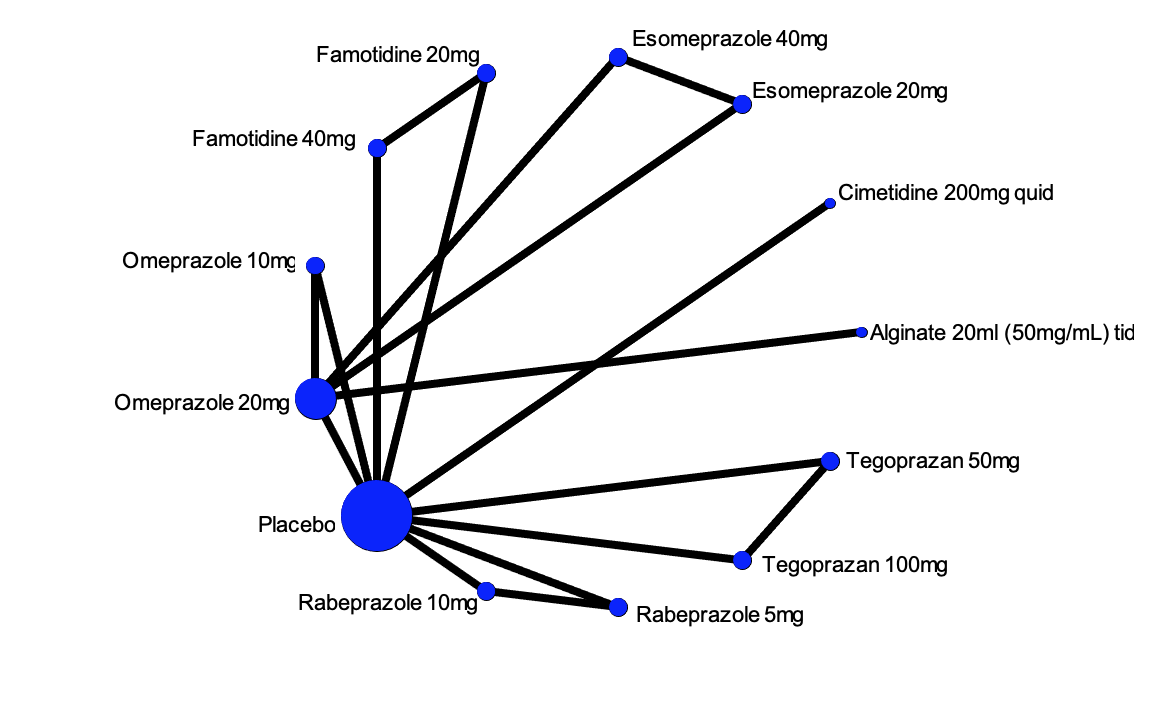
**

**Supplementary Figure 2. Network Plot for Failure to Achieve Adequate Relief Between ≥2 and <4 Weeks of Treatment.**

**
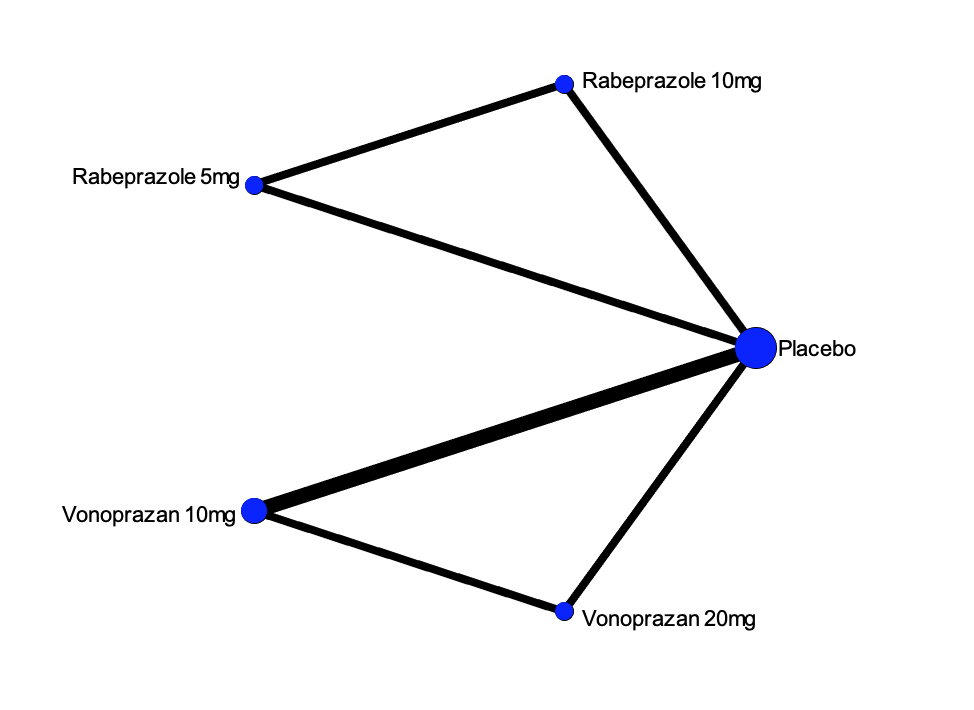
**

**Supplementary Figure 3. Network Meta-analysis of Likelihood of Failure to Achieve Complete Relief at ≥4 Weeks of Treatment.**

1. **Network Plot for Failure to Achieve Complete Relief at ≥4 Weeks of Treatment.**

**
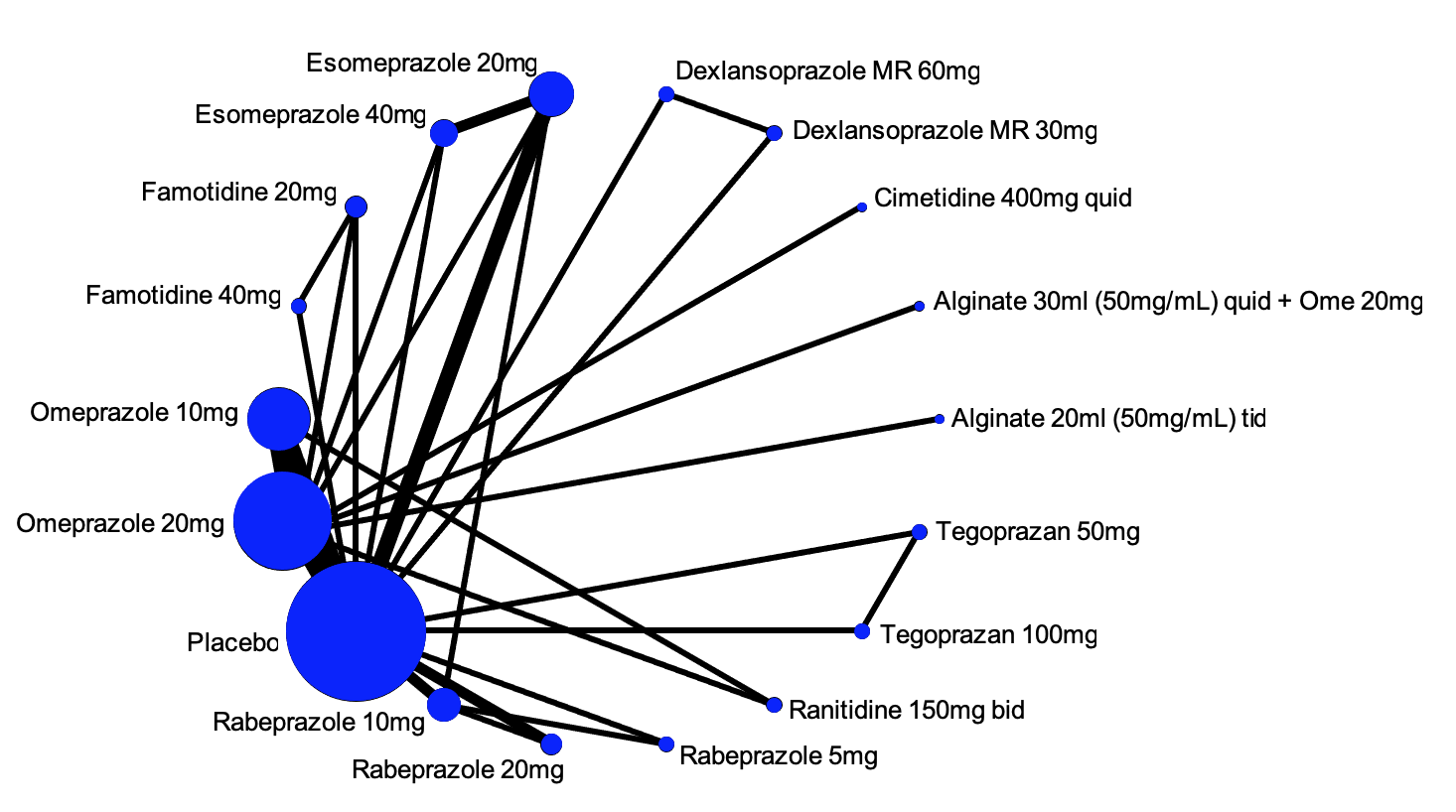
**

1. **Funnel Plot for Failure to Achieve Complete Relief at ≥4 Weeks of Treatment.**

**Supplementary Figure 4. Network Meta-analysis of Likelihood of Failure to Achieve Adequate Relief at ≥4 Weeks of Treatment.**

1. **Network Plot for Failure to Achieve Adequate Relief at ≥4 Weeks of Treatment.**

**
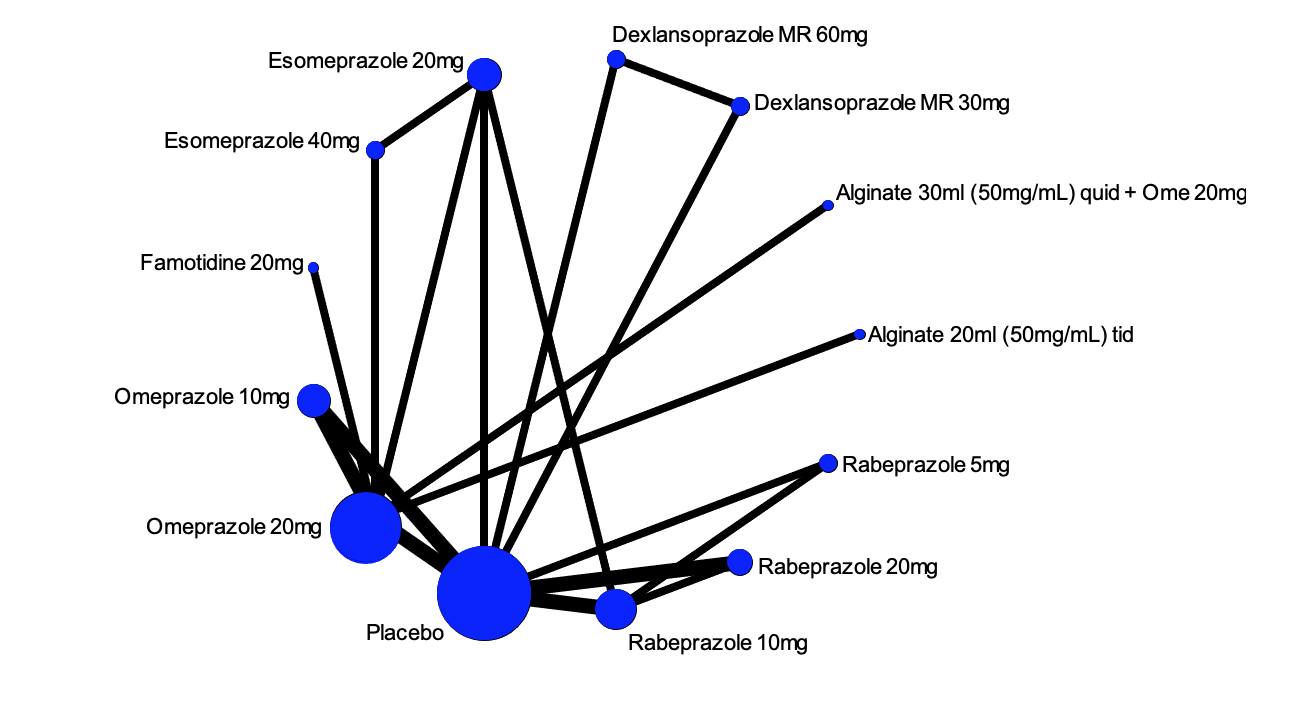
**

1. **Funnel Plot for Failure to Achieve Adequate Relief at ≥4 Weeks of Treatment.**

**Supplementary Figure 5. Network Meta-analysis of Likelihood of Adverse Events.**

1. **Network Plot for Any Adverse Event.**

**
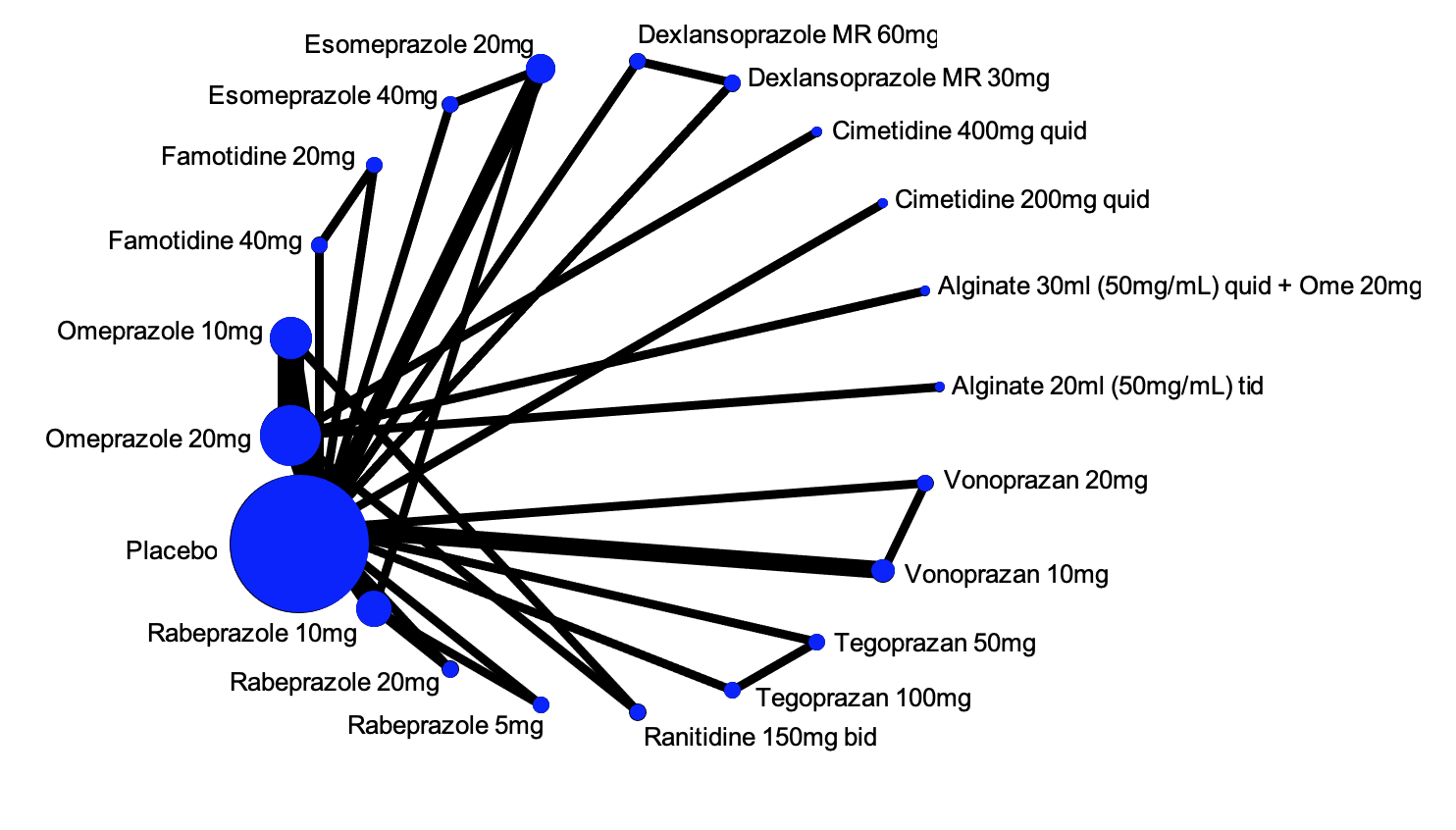
**

1. **Funnel Plot for Any Adverse Event.**

1. **Forest Plot for Any Adverse Event.**

**
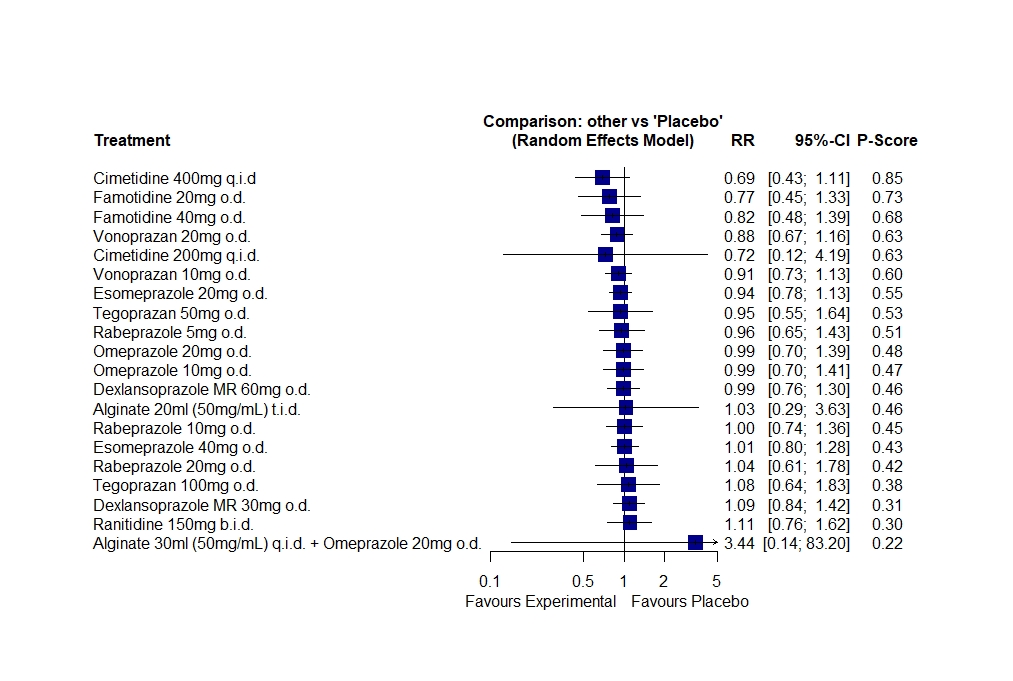
**

Note: Treatments are reported in order of efficacy ranking according to P-score.

The P-score is the probability of each treatment being ranked as best in terms of safety in the network.

**Supplementary Figure 6. Network Meta-analysis of Likelihood of Withdrawal Due to Adverse Events.**

1. **Network Plot for Withdrawal Due to Adverse Events.**

**
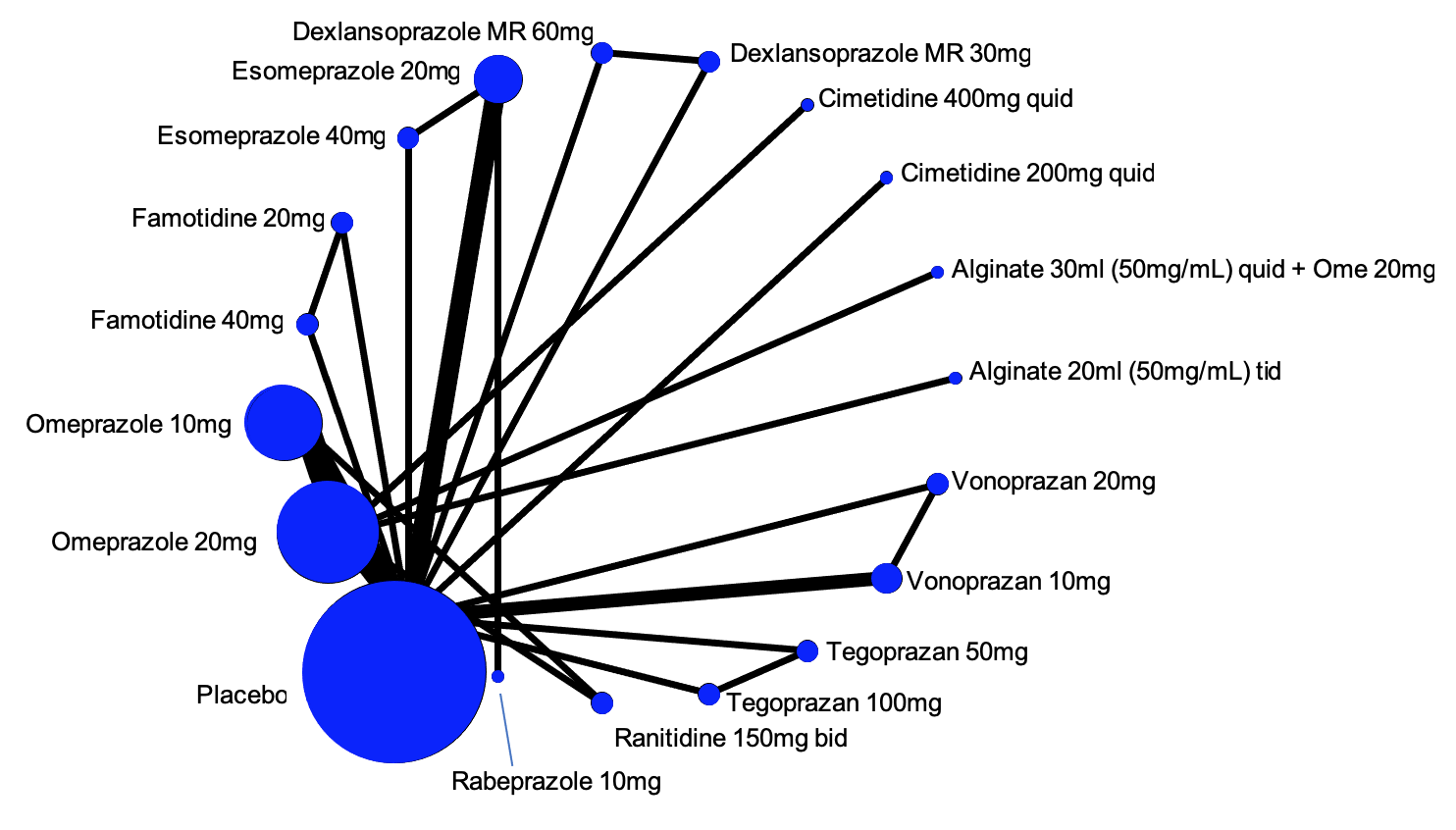
**

1. **Funnel Plot for Withdrawal Due to Adverse Events.**

1. **Forest Plot for Withdrawal Due to Adverse Events.**

**
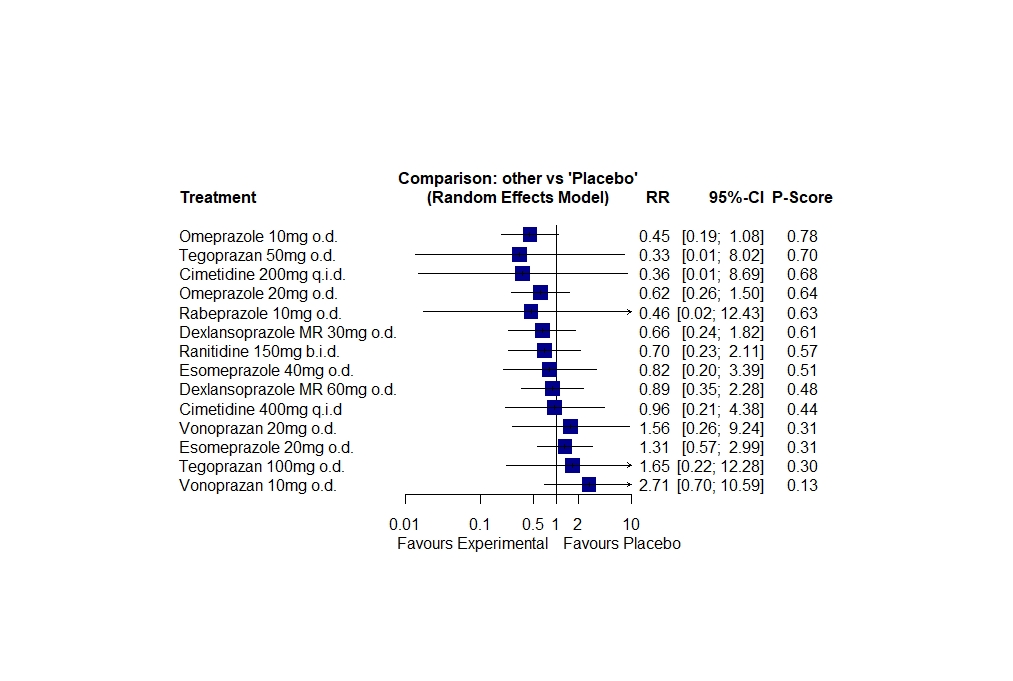
**

Note: Treatments are reported in order of efficacy ranking according to P-score.

The P-score is the probability of each treatment being ranked as best in terms of safety in the network.

**REFERENCES**

1. Riemann JF, Hobel W. Cimetidine suspension in patients with stage 0 gastro-oesophageal reflux disease. Aliment Pharmacol Ther 1991;5:191–197.

2. Robinson M, Decktor DL, Stone RC, et al. Famotidine (20 mg) b.d. relieves gastrooesophageal reflux symptoms in patients without erosive oesophagitis. Famotidine/GERD Investigation Group. Aliment Pharmacol Ther 1991;5:631–643.

3. Bate CM, Griffin SM, Keeling PWN, et al. Reflux symptom relief with omeprazole in patients without unequivocal oesophagitis. Aliment Pharmacol Ther 1996;10:547–555.

4. Bate CM, Green JRB, Axon ATR, et al. Omeprazole is more effective than cimetidine for the relief of all grades of gastro-oesophageal reflux disease-associated heartburn, irrespective of the presence or absence of endoscopic oesophagitis. Aliment Pharmacol Ther 1997;11:755–763.

5. Lind T, Havelund T, Carlsson R, et al. Heartburn without oesophagitis: efficacy of omeprazole therapy and features determining therapeutic response. Scand J Gastroenterol 1997;32:974–979.

6. Venables TL, Newland RD, Patel AC, et al. Omeprazole 10 milligrams once daily, omeprazole 20 milligrams once daily, or ranitidine 150 milligrams twice daily, evaluated as initial therapy for the relief of symptoms of gastro-oesophageal reflux disease in general practice. Scand J Gastroenterol 1997;32:965–973.

7. Carlsson R, Dent J, Watts R, et al. Gastro-oesophageal reflux disease in primary care: an international study of different treatment strategies with omeprazole. International GORD Study Group - PubMed. Eur J Gastroenterolol Hepatolo 1998;10.2:119–24.

8. Richter JE, Peura D, Benjamin SB, et al. Efficacy of omeprazole for the treatment of symptomatic acid reflux disease without esophagitis. Arch Intern Med 2000;160:1810–1816.

9. Miner P, Orr W, Filippone J, et al. Rabeprazole in nonerosive gastroesophageal reflux disease: a randomized placebo-controlled trial. Am J Gastroenterol 2002;97:1332–1339.

10. Katz PO, Castell DO, Levine D. Esomeprazole resolves chronic heartburn in patients without erosive oesophagitis. Aliment Pharmacol Ther 2003;18:875–882.

11. Armstrong D, Talley NJ, Lauritsen K, et al. The role of acid suppression in patients with endoscopy-negative reflux disease: the effect of treatment with esomeprazole or omeprazole. Aliment Pharmacol Ther 2004;20:413–421.

12. Fock KM, Teo EK, Ang TL, et al. Rabeprazole vs esomeprazole in non-erosive gastro-esophageal reflux disease: A randomized, double-blind study in urban Asia. World J Gastroenterol 2005;11:3091.

13. Fujiwara Y, Higuchi K, Nebiki H, et al. Famotidine vs. omeprazole: a prospective randomized multicentre trial to determine efficacy in non-erosive gastro-oesophageal reflux disease. Aliment Pharmacol Ther 2005;21 Suppl 2:10–18.

14. Kahrilas PJ, Miner P, Johanson J, et al. Efficacy of rabeprazole in the treatment of symptomatic gastroesophageal reflux disease. Dig Dis Sci 2005;50:2009–2018.

15. Uemura N, Inokuchi H, Serizawa H, et al. Efficacy and safety of omeprazole in Japanese patients with nonerosive reflux disease. J Gastroenterol 2008;43:670–678.

16. Fass R, Chey WD, Zakko SF, et al. Clinical trial: the effects of the proton pump inhibitor dexlansoprazole MR on daytime and nighttime heartburn in patients with non-erosive reflux disease. Aliment Pharmacol Ther 2009;29:1261–1272.

17. Kinoshita Y, Ashida K, Hongo M. Randomised clinical trial: a multicentre, double-blind, placebo-controlled study on the efficacy and safety of rabeprazole 5 mg or 10 mg once daily in patients with non-erosive reflux disease. Aliment Pharmacol Ther 2011;33:213–224.

18. Tan VPY, Wong WM, Cheung TK, et al. Treatment of non-erosive reflux disease with a proton pump inhibitor in Chinese patients: a randomized controlled trial. J Gastroenterol 2011;46:906–912.

19. Manabe N, Haruma K, Ito M, et al. Efficacy of adding sodium alginate to omeprazole in patients with nonerosive reflux disease: a randomized clinical trial. Dis esophagus Off J Int Soc Dis Esophagus 2012;25:373–380.

20. Chiu CT, Hsu CM, Wang CC, et al. Randomised clinical trial: sodium alginate oral suspension is non-inferior to omeprazole in the treatment of patients with non-erosive gastroesophageal disease. Aliment Pharmacol Ther 2013;38:1054–1064.

21. Kinoshita Y, Sakurai Y, Shiino M, et al. Evaluation of the Efficacy and Safety of Vonoprazan in Patients with Nonerosive Gastroesophageal Reflux Disease: A Phase III, Randomized, Double-Blind, Placebo-Controlled, Multicenter Study. Curr Ther Res Clin Exp 2016;81–82:1.

22. Kinoshita Y, Sakurai Y, Takabayashi N, et al. Efficacy and Safety of Vonoprazan in Patients With Nonerosive Gastroesophageal Reflux Disease: A Randomized, Placebo-Controlled, Phase 3 Study. Clin Transl Gastroenterol 2019;10.

23. Kim SH, Cho KB, Chun HJ, et al. Randomised clinical trial: comparison of tegoprazan and placebo in non-erosive reflux disease. Aliment Pharmacol Ther 2021;54:402–411.
